# Comparative study between the effect of Nissen fundoplication and Toupet fundoplication on postoperative manometry findings. A randomized control trial study

**DOI:** 10.1007/s00464-025-12112-6

**Published:** 2025-08-26

**Authors:** Edward Atef Youssef Gadalla, Mohamed Ezzat el Serafy, Ayman Hossam El din abd El Monaem, Enaam Ali El Sayed, Ashraf Kamal Mohamad Abdalla

**Affiliations:** 1 General Surgery Department, Faculty of Medicine, Ain Shams University, Cairo, Egypt; 2 Tropical Medicine Department, Faculty of Medicine, Ain Shams University, Cairo, Egypt

**Keywords:** GERD, Toupet fundoplication, Nissen fundoplication, High-resolution manometry, Randomized controlled trial, Anti-reflux surgery

## Abstract

**Background:**

Gastro-esophageal reflux disease (GERD) impairs quality of life and may require surgery when medical treatment fails. Laparoscopic Nissen fundoplication (LNF) is the traditional gold-standard, whereas laparoscopic Toupet fundoplication (LTF) may provide comparable reflux control with fewer motility-related side-effects.

**Methods:**

We conducted a single-center, parallel-group, randomized controlled trial (ClinicalTrials.gov identifier: NCT05432109) at Ain Shams University Hospitals. Twenty adults with medically refractory GERD were randomized (1:1) to LNF or LTF. Primary outcome was change in lower esophageal sphincter (LES) pressure measured by high-resolution manometry (HRM) 6 weeks post-operatively. Secondary outcomes included distal contractile integral (DCI), largest break size, distal latency (DL), hiatus hernia resolution, GERD Health-Related Quality of Life (GERD-HRQL) score, and adverse events. Analyses followed CONSORT guidelines and utilized an intention-to-treat approach.

**Results:**

Both procedures significantly increased median LES pressure (LNF: + 12 mmHg; LTF: + 10 mmHg, p < 0.001 each). LTF produced greater improvement in largest break size (− 1.60 cm vs. − 1.00 cm; p = 0.013) and GERD-HRQL (median reduction − 3 vs. − 2; p = 0.019). DCI rose more after LTF (+ 710 vs. + 225 mmHg s cm; p = 0.051). Early dysphagia occurred in 40% of LNF versus 10% of LTF patients, while gas-bloat syndrome occurred in 30% vs. 0% (p = 0.07 and 0.04, respectively). No serious adverse events were observed.

**Conclusions:**

LTF provided equivalent reflux control, superior motility preservation, and fewer early obstructive symptoms compared with LNF. LTF should be considered the preferred anti-reflux procedure, particularly in patients with borderline esophageal motility. Larger multicenter trials with longer follow-up are warranted.

**Graphical abstract:**

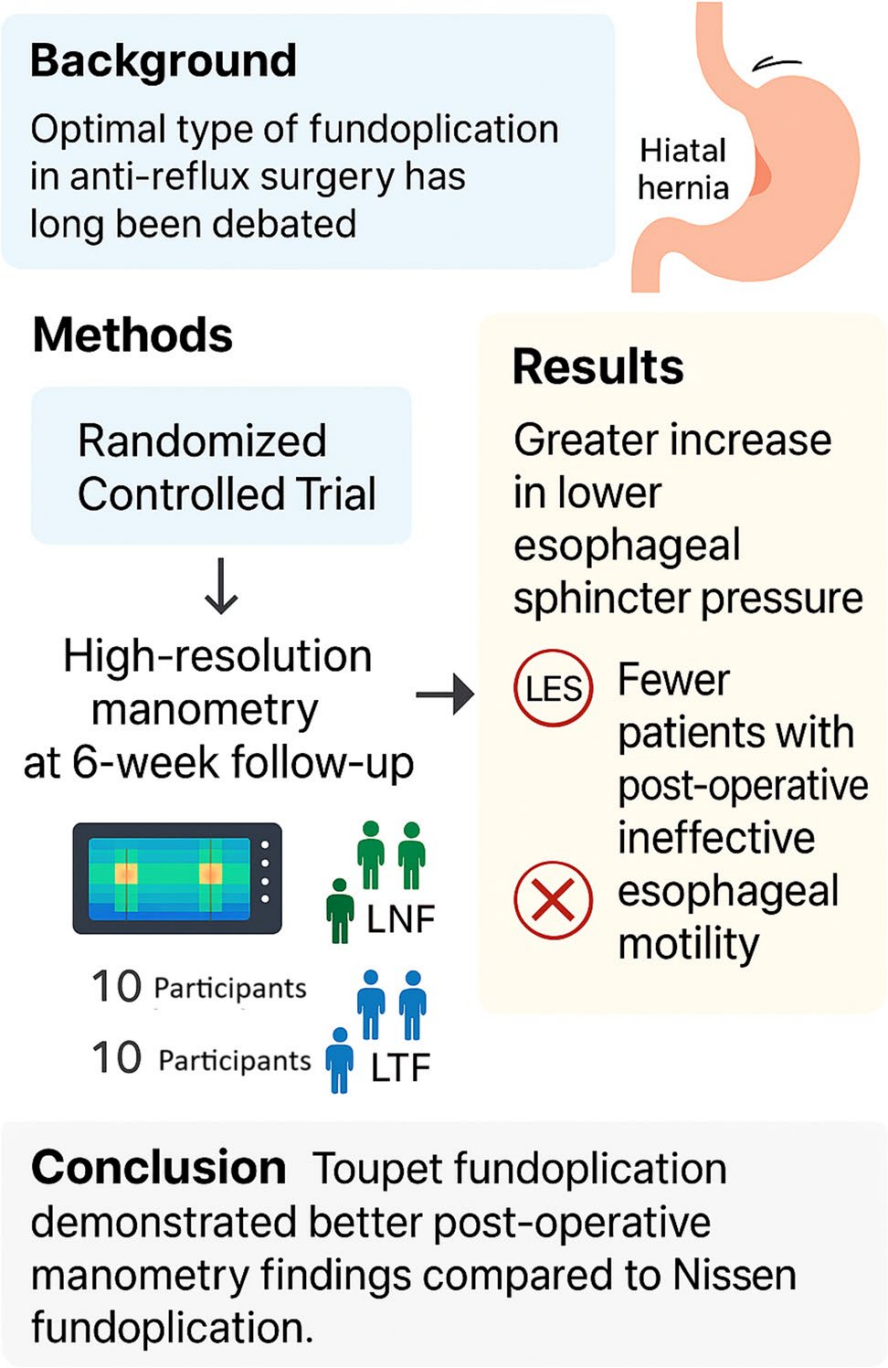

## Background

Gastro-esophageal reflux disease (GERD) affects approximately 10–20% of adults in Western populations and up to 12% of Middle Eastern adults, causing both typical symptoms (heartburn, regurgitation) and extra-esophageal manifestations. It also predisposes to Barrett’s esophagus and esophageal adenocarcinoma [1, 2]. Anti-reflux surgery is recommended when optimal proton pump inhibitor therapy fails, is not tolerated, or when complications such as Barrett’s esophagus or volume reflux develop [3].

Laparoscopic Nissen fundoplication (LNF), a 360° total wrap, provides durable reflux control but is associated with postoperative dysphagia and gas-bloat syndrome [4, 5]. Laparoscopic Toupet fundoplication (LTF), a 270° posterior partial wrap, may reduce these adverse effects while maintaining comparable efficacy [6–8]. High-resolution manometry (HRM) enables objective assessment of lower esophageal sphincter (LES) function and esophageal body motility, and is increasingly used to guide surgical decision-making [9].

Randomized data directly comparing HRM outcomes following LNF and LTF remain limited [6, 10, 11]. We therefore conducted a prospective randomized trial to compare postoperative HRM parameters and symptom relief between LNF and LTF.

## Patients and methods

This prospective randomized controlled trial was conducted at Ain Shams University Hospitals to evaluate the outcomes of laparoscopic Nissen versus Toupet fundoplication in patients with medically refractory GERD. The study enrolled 20 adult patients who were randomly allocated to receive either a laparoscopic Nissen fundoplication (360° wrap) or Toupet fundoplication (270° posterior wrap) using a computer-generated randomization sequence with maintained allocation concealment.

### Inclusion and exclusion criteria

Patients were eligible for inclusion if they were aged 18 to 65 years and had a confirmed diagnosis of GERD with low lower esophageal sphincter (LES) pressure, as measured by high-resolution manometry (HRM). Additional criteria included persistent GERD symptoms despite at least 12 weeks of maximal medical therapy, intolerance or non-compliance with medical therapy, and esophageal motility of at least 30% to ensure sufficient peristalsis postoperatively. Based on preoperative HRM using Chicago Classification v4.0, all enrolled patients demonstrated ineffective esophageal motility (IEM). No cases of absent contractility were identified, and patients with such findings were excluded. Thus, both groups had 100% IEM and 0% normal motility at baseline.

We note that HRM serves distinct diagnostic roles in this study—assessing both esophageal motility patterns (according to the Chicago Classification) and hiatal hernia status (based on LES–crural diaphragm separation). These assessments were conducted and interpreted independently in accordance with established HRM methodology.

Exclusion criteria included esophageal motility disorders with peristalsis less than 30%, morbid obesity (BMI > 35 kg/m^2^), history of prior upper abdominal surgery, a severely shortened esophagus, extremes of age (< 18 or > 65 years), and patients classified as ASA III, IV, or V, indicating poor general fitness for surgery.

All hiatal hernias included in this study were primary. Patients with recurrent hiatal hernias or any history of prior anti-reflux or upper abdominal surgery were excluded to eliminate confounding factors related to surgical failure or altered anatomy.

### Randomization and study design

Patients were randomly assigned in a 1:1 ratio to undergo either laparoscopic Nissen fundoplication (LNF) or laparoscopic Toupet fundoplication (LTF) through a computer-generated block sequence (blocks of four) prepared by an independent statistician. Allocation was concealed in sequentially numbered, opaque envelopes opened intra-operatively after diagnostic laparoscopy, yielding two equal groups of 10 patients each (N Group and T Group).

**Fig. 1:**
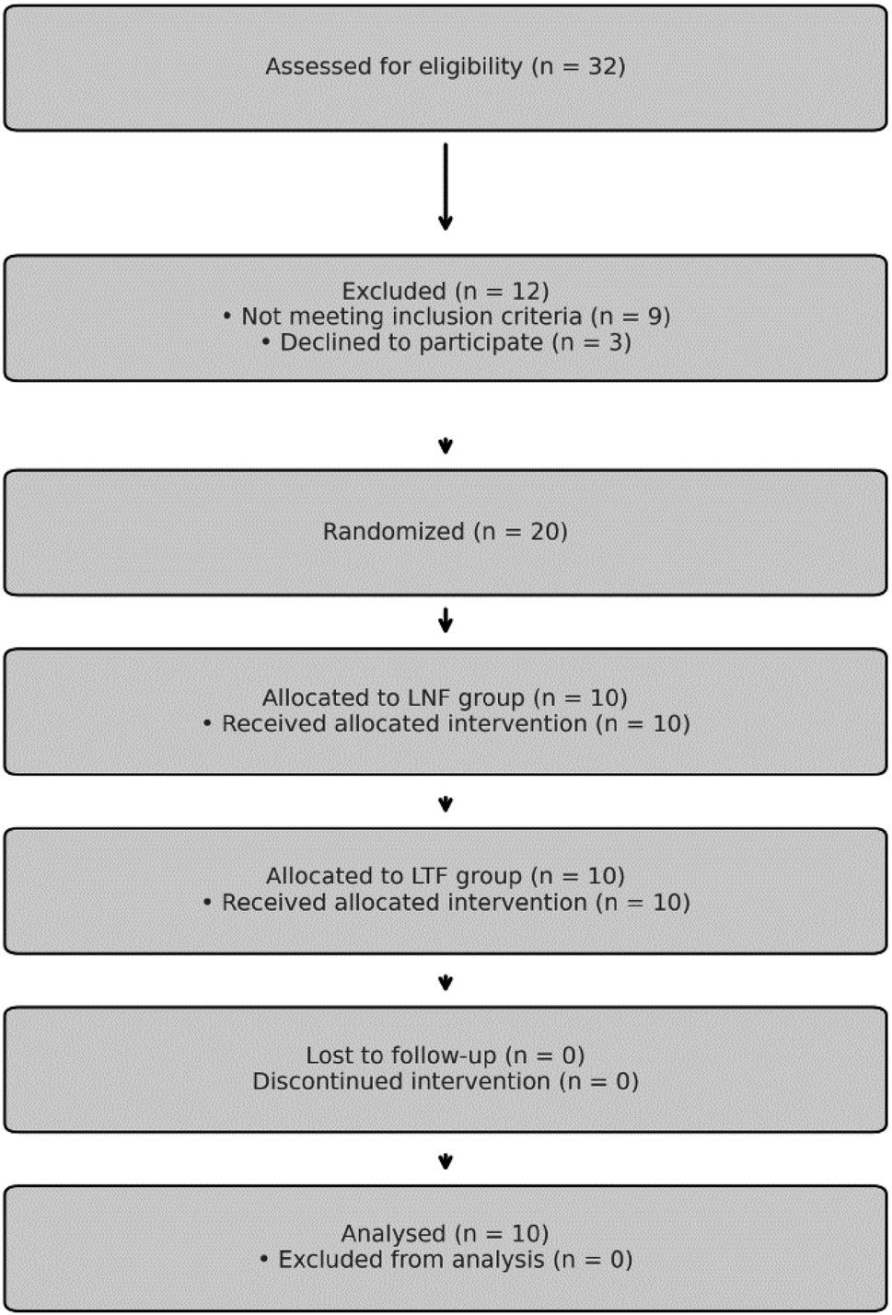
CONSORT flow diagram

Figure 1 provides the CONSORT diagram outlining patient recruitment, allocation, and follow-up.

The trial followed a double-blind design: while the operating surgeon was necessarily unblinded, all patients, outcome assessors, and data analysts remained blinded to the group assignment. The surgeons had no role in postoperative outcome evaluation or statistical analysis.

### Sample size

Assuming a mean LES pressure increase of 10 ± 4 mmHg after LNF and 13 ± 3 mmHg after LTF [13], 8 participants per arm provided 80% power (α = 0.05, two tailed). To allow for attrition, 10 patients were enrolled per group.

### Preoperative evaluation

All patients underwent comprehensive preoperative evaluation, including detailed symptom assessment, PPI usage history, and identification of extra-esophageal manifestations such as chronic cough or aspiration. Diagnostic investigations included barium swallow, upper GI endoscopy, and high-resolution manometry (HRM) to assess anatomical integrity, mucosal condition, and esophageal motility, with GERD-HRQL questionnaires administered to measure quality of life. Surgery followed a standardized laparoscopic protocol with 2–3 cm fundoplication wraps and crural repair, performed by experienced surgeons. Postoperative assessments at 6 weeks included repeat HRM and GERD-HRQL scoring, LES pressure and motility outcomes.

Hiatal hernia status and size were assessed in all patients preoperatively using high-resolution manometry (HRM) and barium swallow imaging. A separation of ≥ 2 cm between the lower esophageal sphincter (LES) and the diaphragmatic impression on HRM was considered diagnostic. Based on this criterion, hiatal hernia was present in 70% of patients in the Nissen group and 80% in the Toupet group. All identified hernias were small sliding hernias (< 3 cm axial length); no large (> 5 cm), paraoesophageal, or mixed-type hernias were included. Median hernia size was comparable between groups (see “Results” section). All patients underwent standardized crural repair, regardless of hernia presence or size.

All patients underwent upper gastrointestinal endoscopy to assess mucosal health and exclude structural pathology. Endoscopic oesophagitis (LA Grade A or B) was identified in 40% of patients. No cases of Barrett’s esophagus were observed, and no biopsies were taken. Patients with suspected Barrett’s or mucosal abnormalities requiring histological evaluation were excluded from the study and referred for appropriate diagnostic follow-up.

All patients had completed at least 12 weeks of standardized maximal-dose PPI therapy (e.g., omeprazole 40 mg twice daily) prior to enrollment. Postoperatively, PPIs were uniformly discontinued on postoperative day one. Patients were instructed to remain off PPIs unless significant symptoms recurred.

### Surgical techniques

#### Laparoscopic Nissen Fundoplication (LNF)

**Fig. 2:**
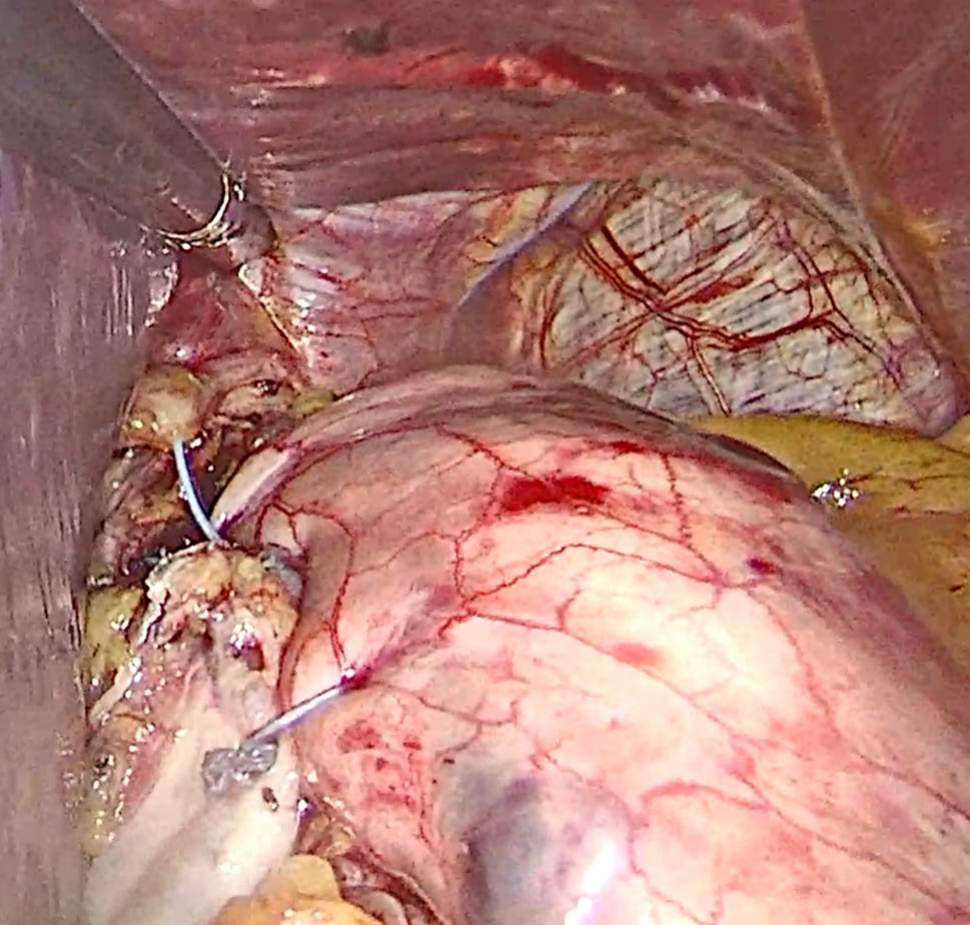
Intraoperative image showing Laparoscopic Nissen Fundoplication (360° wrap)

In this procedure, a 360° wrap of the gastric fundus was created around the esophagus to reinforce the LES. The surgery was performed under general anesthesia, with the patient placed in the modified lithotomy position. A total of five or six laparoscopic ports were inserted to allow dissection and mobilization of the fundus. The short gastric vessels were divided to ensure adequate fundic mobilization. The crural diaphragmatic repair was performed using non-absorbable ethibond sutures, followed by the creation of a 1.5 to 2 cm loose wrap around the esophagus, anchored with three interrupted sutures. A 50F esophageal bougie was routinely used to prevent excessive tightness of the wrap. An example of the complete warp is shown in Fig. 2.

#### Laparoscopic Toupet Fundoplication (LTF)

**Fig. 3:**
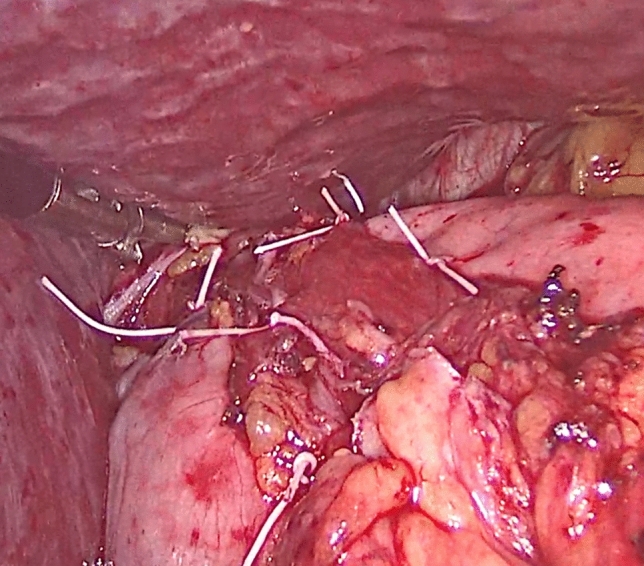
Intraoperative image showing Laparoscopic Toupet Fundoplication (270° posterior wrap)

The 270° posterior fundoplication technique was used in this group, with the goal of maintaining LES competence while reducing postoperative dysphagia. The procedure followed the same laparoscopic approach as Nissen fundoplication, with the gastric fundus being positioned posteriorly around the esophagus. Two to three sutures securing the wrap to the esophagus. An example of the partial wrap is shown in Fig. 3.

#### Post Operative follow up and evaluation

Patients were followed for a period of 6 weeks postoperatively. Symptom resolution was assessed based on improvements in heartburn, regurgitation, and chest pain, along with the occurrence of dysphagia and gas-bloating syndrome. A postoperative HRM was conducted after 6 weeks to evaluate changes in LES pressure and esophageal motility compared to preoperative values.

All operations in both groups were performed by a single senior consultant surgeon experienced in minimally invasive foregut surgery. This approach ensured consistency in surgical technique and minimized inter-operator variability across both arms of the trial.

### Statistical analysis

All statistical analyses were performed using GraphPad Prism® version 10.0 with an intention-to-treat approach. Continuous variables were analyzed using non-parametric tests due to the study's sample size characteristics. The Mann–Whitney U test was employed for between-group comparisons of continuous variables, including high-resolution manometry (HRM) metrics and GERD-Health Related Quality of Life (GERD-HRQL) scores. For categorical data analysis, Fisher's exact test was applied to 2 × 2 contingency tables, while the chi-square (χ^2^) test was used for larger categorical variable comparisons, particularly for complication rates and other discrete outcomes.

A two-tailed p-value of < 0.05 was established as the threshold for statistical significance throughout all analyses, indicating meaningful differences between the Nissen and Toupet fundoplication approaches. The study design incorporated both within-group (preoperative vs. postoperative) and between-group (Nissen vs. Toupet) comparisons to comprehensively evaluate surgical outcomes. All statistical procedures were conducted in accordance with the study protocol, which received Institutional Review Board approval prior to implementation. Written informed consent was obtained from all participants before enrollment, ensuring strict adherence to ethical guidelines and regulatory requirements throughout the trial duration.

### Ethical considerations

This study was approved by the Research Ethics Committee of Ain Shams University. Informed consent was obtained from all patients before enrollment, ensuring that they fully understood the study objectives and potential risks. Patients were given the right to withdraw from the study at any stage without affecting their access to standard medical care. All data collected were handled with strict confidentiality, ensuring compliance with ethical research guidelines.

## Results

**Fig. 4:**
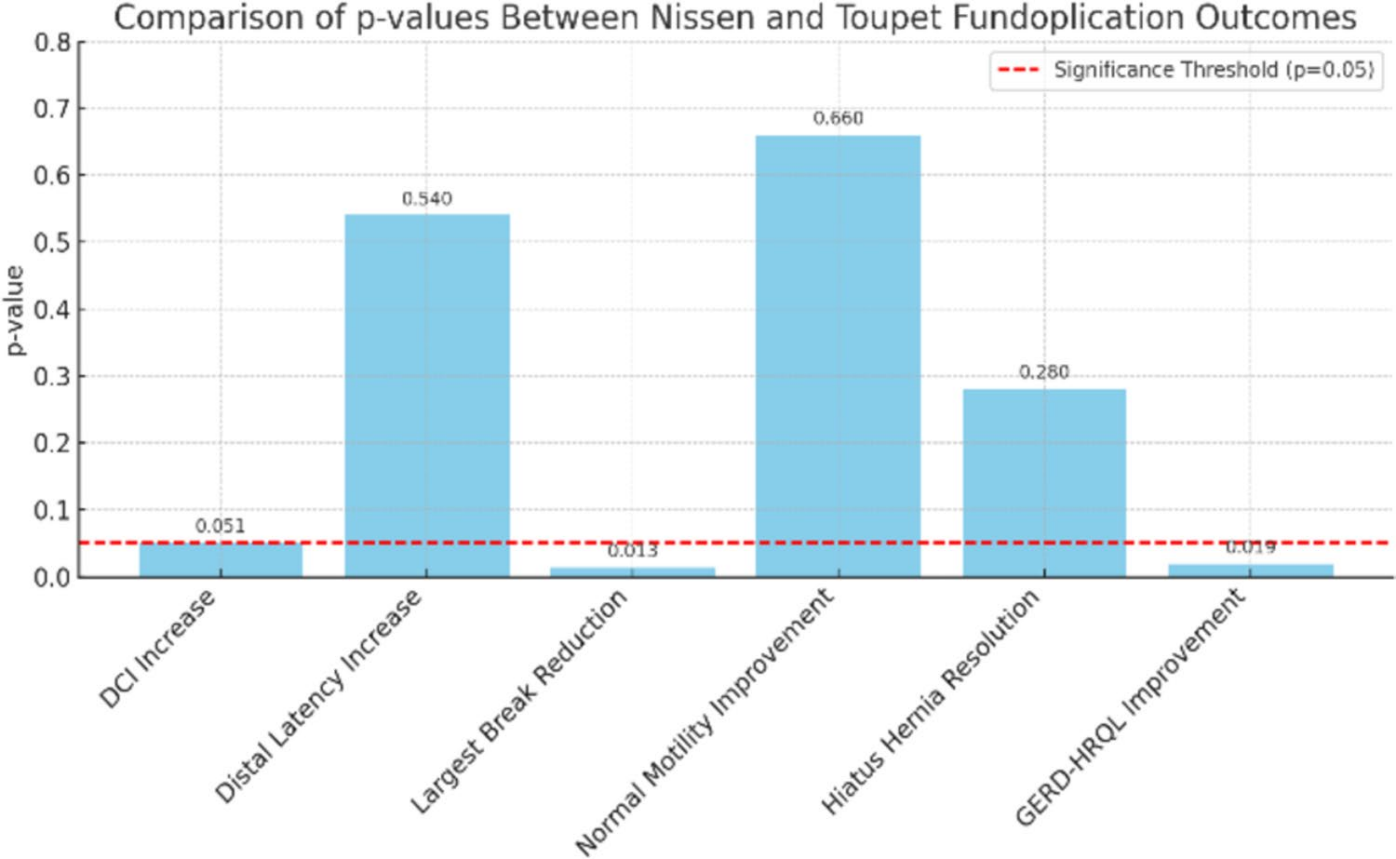
Comparative statistical significance (p-values) of key manometric and clinical outcomes between Nissen and Toupet fundoplication

Both Nissen and Toupet fundoplication produced significant improvements in esophageal high-resolution manometry (HRM) metrics. Figure 4 summarises the p-values for the primary and secondary outcomes, highlighting statistically significant improvements in break size and GERD-HRQL in the Toupet group.

In the Nissen group, the median DCI increased from 650 [561–741] mmHg s cm pre-operatively to 875 [763–1107] post-operatively (p = 0.049). In the Toupet group, the median DCI increased from 440 [363–505] to 1150 [688–1338] mmHg s cm (p = 0.002). A larger DCI increase was noted with Toupet (p = 0.051).

**Fig. 5:**
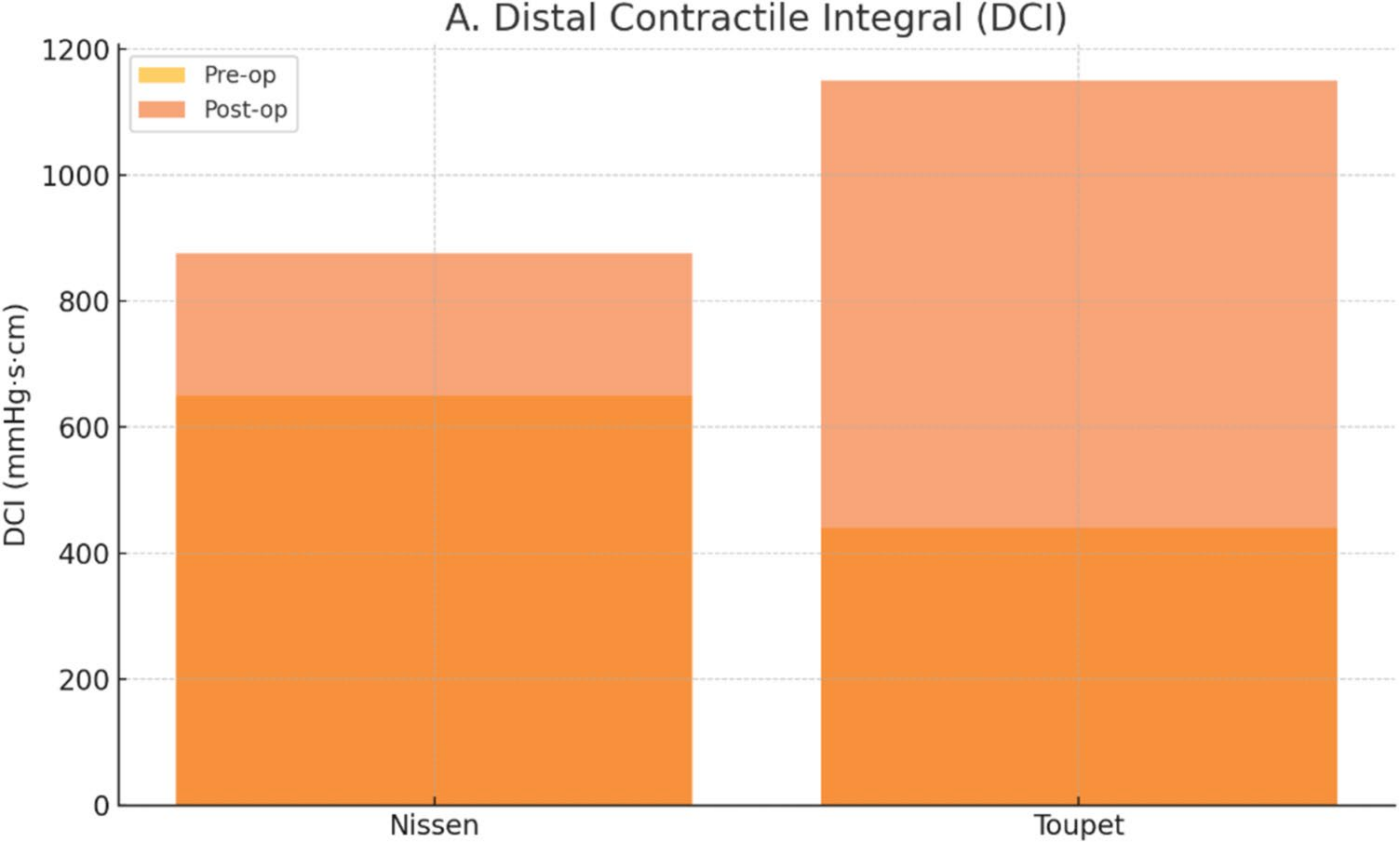
Improvement in distal contractile integral (DCI) following fundoplication surgery

Figure 5 visually illustrates these changes in DCI, showing a greater postoperative increase in the Toupet group.

Regarding distal latency, the Nissen group showed prolongation from 6.35 [6.08–6.57] to 7.30 [7.00–7.73] s (p = 0.002), while the Toupet group showed an increase from 6.10 [5.58–6.43] to 7.50 [7.23–7.95] s (p = 0.002). However, the difference in distal latency improvement between groups was not statistically significant (p = 0.54).

**Fig. 6:**
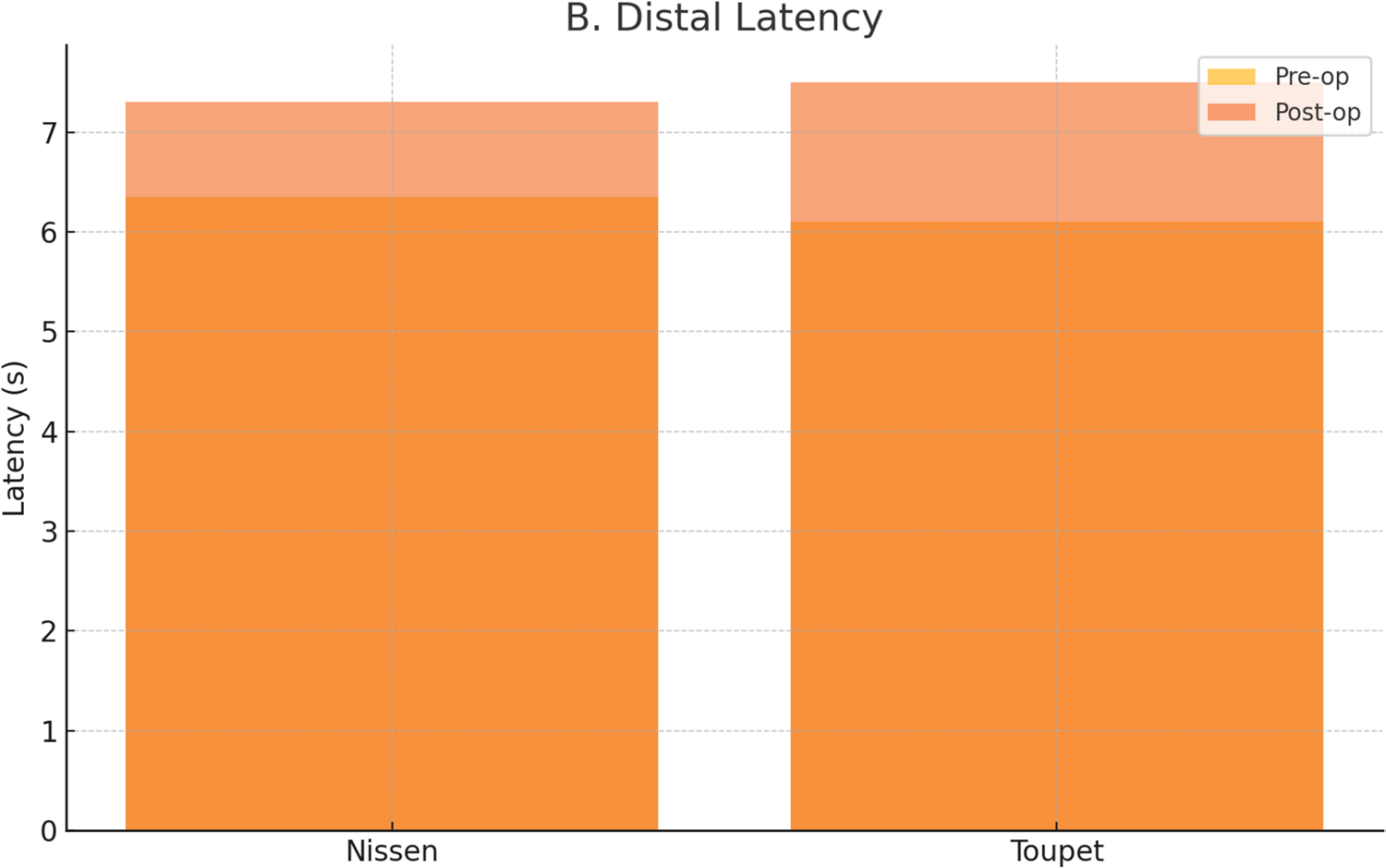
Distal latency changes pre- and postoperatively in Nissen and Toupet groups

Figure 6 depicts the changes in distal latency before and after surgery, confirming similar trends in both groups.

The largest break size decreased in both groups, with Nissen patients showing a reduction from 1.95 [1.50–2.28] cm to 0.95 [0.55–1.43] cm (p = 0.049) and Toupet patients from 2.70 [2.13–3.00] cm to 1.10 [0.70–1.68] cm (p = 0.002). The greater reduction was observed with Toupet (p = 0.013).

**Fig. 7:**
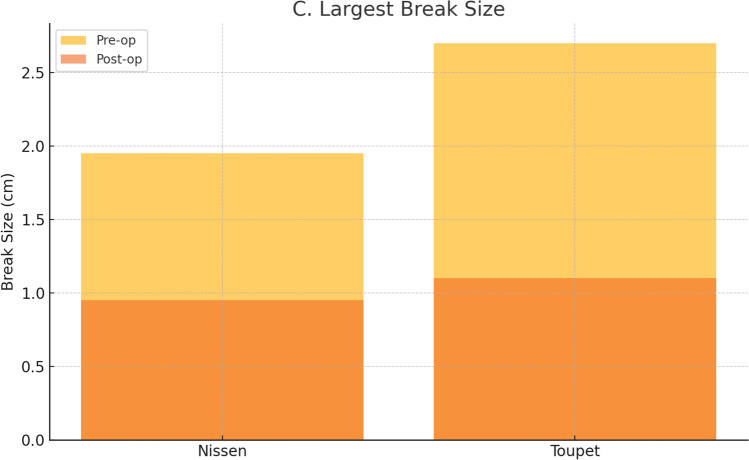
Reduction in largest break size following Nissen and Toupet fundoplication

Figure 7 presents these reductions graphically, highlighting the superior improvement in break size following Toupet fundoplication.

In the Nissen group, normal motility increased from 0% pre-operatively to 40% post-operatively, while ineffective motility persisted in 50% of patients. In the Toupet group, normal motility increased from 0 to 60%, with a more marked reduction in ineffective motility to 30% post-operatively. Although the trend favored Toupet, the difference between groups was not statistically significant (p = 0.66).

Hiatus hernia was present in 70% of Nissen patients pre-operatively, resolving completely in only 14%. In the Toupet group, hiatus hernia was present in 80% of patients and resolved in 50%. Although the trend favored Toupet, the difference was not statistically significant (p = 0.28). We emphasize that these resolution rates reflect HRM-based detection of LES–crural separation rather than anatomical imaging. Factors such as postoperative oedema and altered tissue compliance may have influenced early manometric interpretation. All patients were asymptomatic and showed significant improvement in GERD-HRQL, suggesting clinically effective anatomical outcomes despite residual HRM separation in some cases.

The median axial length of hiatal hernia was 2.3 cm [1.5–3.0] in the Nissen group and 2.5 cm [1.8–3.2] in the Toupet group (p = 0.67), confirming baseline comparability of anatomical severity between the cohorts.

Both groups demonstrated significant improvements in GERD-HRQL scores. The Nissen group improved from a median score of 3 [2–4] to 1 [1, 2] (p = 0.002), while the Toupet group improved from 4 [3–5] to 1 [0–1] (p = 0.002). A significantly greater reduction in GERD-HRQL scores was achieved in the Toupet group compared to the Nissen group (p = 0.019).

**Fig. 8:**
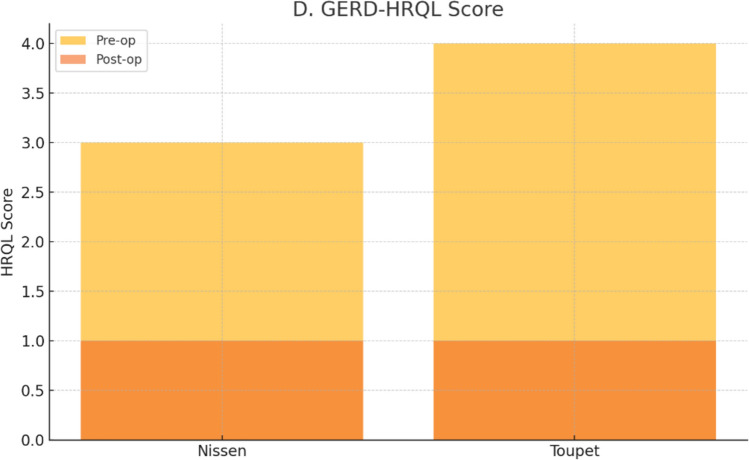
Pre- and postoperative GERD-health related quality of life (GERD-HRQL) score changes

This is further demonstrated in Fig. 8, which shows the marked improvement in quality-of-life scores, especially in the Toupet group.

Postoperative assessment of hiatal hernia resolution was based exclusively on high-resolution manometry (HRM), using separation of the lower esophageal sphincter (LES) and crural diaphragm pressure zones as a diagnostic marker. A separation of greater than 2 cm was considered consistent with a hiatal hernia. We acknowledge that this technique, while objective and non-invasive, may have limited sensitivity in accurately detecting subtle anatomical repairs in the early postoperative period. No endoscopy or barium imaging was routinely performed during the 6-week follow-up phase.

Table 1 presents the baseline characteristics of patients in the Nissen and Toupet groups. This includes demographic data (age, sex, BMI), preoperative HRM metrics (LES pressure, DCI, distal latency, break size), hiatal hernia status, and GERD-Health Related Quality of Life (GERD-HRQL) scores. Statistical comparison of these variables confirms adequate balance between groups at baseline, supporting the internal validity of the randomisation process.

**Table 1:** Baseline patient characteristics

Parameter	Nissen Group	Toupet Group	p-value
Age (years)	42.3 ± 11.2	44.1 ± 10.8	0.68
Sex (M/F)	6 / 4	5 / 5	0.65
BMI (kg/m^2^)	27.1 ± 2.5	26.8 ± 2.7	0.74
LES pressure (mmHg)	8.5 ± 3.2	8.7 ± 3.4	0.85
DCI (mmHg s cm)	650 [561–741]	440 [363–505]	0.07
Distal latency (s)	6.35 [6.08–6.57]	6.10 [5.58–6.43]	0.41
Largest break size (cm)	1.95 [1.50–2.28]	2.70 [2.13–3.00]	0.04
Hiatus hernia presence (%)	70%	80%	0.63
GERD-HRQL score	3 [2–4]	4 [3–5]	0.12

Table 2 summarizes the key postoperative outcomes between the two groups, including changes in HRM metrics (LES pressure, DCI, distal latency, break size), GERD-HRQL score improvement, and clinical endpoints such as postoperative dysphagia and gas-bloat syndrome. Between-group effect sizes and 95% confidence intervals are reported to aid interpretation of clinical and statistical significance.

**Table 2:** Postoperative outcomes with effect sizes and confidence intervals

Parameter	Nissen	Toupet	Between-group difference	95% CI	p-value
LES pressure (mmHg)	+ 12	+ 10	− 2	[− 5.4, + 1.4]	0.33
DCI (mmHg s cm)	+ 225	+ 710	+ 485	[+ 350, + 620]	0.051
Distal latency (s)	+ 0.95	+ 1.40	+ 0.45	[− 0.22, + 1.12]	0.54
Largest break size (cm)	− 1.00	− 1.60	− 0.60	[− 1.10, − 0.10]	0.013
GERD-HRQL score	− 2	− 3	− 1	[− 1.8, − 0.2]	0.019
Normal motility (%)	40%	60%	+ 20%	N/A	0.66
Gas bloat (%)	30%	0%	− 30%	N/A	0.04
Early dysphagia (%)	40%	10%	− 30%	N/A	0.07

## Discussion

Gastroesophageal reflux disease (GERD) is highly prevalent and exerts a substantial negative impact on patients’ quality of life [1]. Surgical intervention, most commonly in the form of fundoplication, is indicated when medical therapy fails [2]. The complete 360° laparoscopic Nissen fundoplication (LNF) has long been regarded as the gold standard, yet accumulating evidence now favors the partial 270° laparoscopic Toupet fundoplication (LTF) for many patients because it appears to deliver comparable reflux control while reducing postoperative dysphagia and preserving esophageal motility [3–5].

The present study directly compared LNF and LTF using high-resolution manometry (HRM) and validated symptom scores (GERD-HRQL). Both procedures effectively restored lower esophageal sphincter (LES) pressure and alleviated reflux symptoms, but LTF achieved superior functional outcomes. In particular, patients who underwent LTF exhibited a significantly greater reduction in largest break size (p = 0.013) and larger improvements in GERD-HRQL scores (p = 0.019). It should be noted, however, that the Toupet group had a slightly higher baseline median break size compared to the Nissen group (2.70 cm vs. 1.95 cm), potentially exaggerating the degree of observed improvement. This imbalance, although unintended, reflects random variation inherent in small sample sizes and does not necessarily imply systematic group mismatch. Nonetheless, this difference should be considered when interpreting the comparative effect of each procedure on this specific manometric parameter. Although the gain in distal contractile integral (DCI) only approached statistical significance (p = 0.051), it nonetheless suggested better contractile recovery with a partial wrap. These findings align with the meta-analysis by Schietroma et al. [11] and the prospective HRM-guided series of Wang et al. [12], both of which reported superior motility preservation and fewer obstructive symptoms after Toupet fundoplication.

Persistent or late-onset dysphagia remains a chief concern after antireflux surgery. In our cohort, early dysphagia and gas bloat were more frequent after LNF, reflecting the tighter outflow resistance created by a 360° wrap. This observation corroborates both historical data [5] and contemporary long-term evidence from MüllerStich et al. [14], who recorded a 19% dysphagia rate 10 years after LNF versus 8% after LTF.

Persistent or late-onset dysphagia remains a chief concern after antireflux surgery. In our cohort, early dysphagia and gas bloat were more frequent after LNF, reflecting the tighter outflow resistance created by a 360° wrap. This observation corroborates both historical data [5] and contemporary long-term evidence from MüllerStich et al. [14], who recorded a 19% dysphagia rate ten years after LNF versus 8% after LTF. Our results support the emerging paradigm that partial wraps, such as Toupet fundoplication, may offer a favourable balance between reflux suppression and luminal clearance, particularly in patients with impaired motility [12].

With respect to hiatus hernia repair, LTF produced a numerically higher—but not statistically significant—rate of hernia resolution, echoing the tension-reduction hypothesis proposed by Koch et al. [13]. Although some authors advocate a complete wrap for large (> 5 cm) hernias, our data reinforce that a carefully crafted partial wrap can achieve durable anatomic correction without the motility penalties of a full wrap.

While our trial confirms the functional advantages of Toupet fundoplication over Nissen fundoplication in patients with impaired motility, these findings should be interpreted in the context of existing prospective randomized trials. Notably, a randomized controlled trial comparing LNF and LTF in 100 patients reported significantly higher rates of postoperative dysphagia and gas-bloat in the LNF group, despite similar reflux control—findings that parallel our results [5]. Another long-term randomized trial with over 20 years of follow-up demonstrated that both techniques remained effective, but patients undergoing Toupet fundoplication reported better swallowing and less bloating, underscoring the long-term functional superiority of partial wraps [9].

More recently, a 10-year randomized follow-up study found persistent dysphagia in 19% of LNF patients versus only 8% in the LTF group, reinforcing concerns about long-term obstruction with full wraps [14]. In terms of HRM outcomes, impaired post-fundoplication motility was more commonly observed after LNF compared to LTF in a randomized cohort, aligning with our findings of greater improvements in DCI and break size with Toupet fundoplication [10]. Additionally, a prospective HRM-guided series demonstrated that tailored partial fundoplication yielded superior esophageal clearance and symptom resolution compared to full wraps [12].

Collectively, these comparisons support the evolving paradigm that partial wraps offer a more favorable balance between reflux control and motility preservation. Our findings complement and extend the results of these prior trials by directly quantifying HRM changes postoperatively and linking them to symptom relief using validated instruments such as the GERD-HRQL score.

Several limitations temper the strength of these conclusions. Most notably, the sample size was modest (n = 10 per group), which limits statistical power and the ability to detect more subtle differences in secondary outcomes. The study was designed as a pilot randomized controlled trial, powered primarily to detect changes in lower esophageal sphincter pressure—our primary endpoint—based on previously reported standard deviations. Nonetheless, the limited cohort size may increase susceptibility to type II error in assessing secondary measures such as DCI, GERD-HRQL, and symptom rates.

Additionally, the follow-up duration of 6 weeks is insufficient to assess long-term surgical durability, including recurrence of reflux, persistent dysphagia, or gas-bloat syndrome. While our goal was to evaluate early postoperative manometric and symptomatic changes, we acknowledge the need for extended follow-up to capture delayed complications and relapse.

Future studies should aim to include larger, multicentre cohorts with longer follow-up periods and standardised imaging reassessment to confirm anatomical durability and validate these preliminary findings. Despite these constraints, our results provide important early evidence supporting the favourable functional profile of Toupet fundoplication in patients with impaired motility.

Another important methodological limitation relates to the timing of randomization, which was performed intraoperatively after diagnostic laparoscopy. While allocation concealment was rigorously maintained through sequentially numbered opaque envelopes and block randomization, we acknowledge that intraoperative assignment may introduce potential for performance bias, particularly in a non-double-blinded surgical setting. Although both procedures were performed by the same experienced consultant using standardized techniques, it is conceivable that intraoperative knowledge of group allocation could subtly influence wrap construction, operative decision-making, or documentation. We believe this risk is mitigated by the use of uniform protocols, but it nonetheless warrants acknowledgement as a possible source of bias.

Furthermore, the single-blind design, in which only the operating surgeon was unblinded, may have introduced bias in subjective outcome measures such as the GERD-Health Related Quality of Life (GERD-HRQL) score. Although patients, outcome assessors, and data analysts remained blinded to intervention group, subjective outcomes remain inherently vulnerable to perception bias. In future studies, a double-blind design with sham blinding techniques (e.g., standardized wound dressings or operative notes) may further reduce this risk and strengthen internal validity, particularly when relying on patient-reported outcome measures.

Conclusion: This randomized trial supports the use of laparoscopic Toupet fundoplication as a safe and effective alternative to Nissen fundoplication for GERD, with superior motility outcomes and fewer early obstructive symptoms. These results reinforce the growing preference for tailored surgical approaches based on manometric profiles.

Both Nissen and Toupet fundoplication remain effective anti-reflux operations, yet the evidence from our cohort and an increasingly robust literature base indicates that Toupet fundoplication (270° posterior wrap) offers more consistent physiological and symptomatic advantages. In our series, Toupet fundoplication yielded superior improvements in esophageal motility, more reliable hiatus-hernia repair, and greater relief of symptoms, while also minimizing the risk of postoperative dysphagia or functional obstruction benefits that are especially valuable in patients with baseline motility impairment. Taken together, these data reinforce the emerging consensus that laparoscopic Toupet fundoplication should often be considered the procedure of choice for many GERD patients in contemporary antireflux practice.

The relatively low rates of hiatal hernia resolution—14% in LNF and 50% in LTF—may appear unexpectedly modest, particularly given the standardised crural repair applied to all patients. However, we believe these figures may be influenced by the inherent limitations of HRM-based diagnosis in the early postoperative period. Oesophageal oedema, altered compliance, or wrap-related tension may affect HRM accuracy. Importantly, despite these findings, all patients demonstrated substantial symptomatic improvement with no clinical signs of anatomical failure at 6 weeks. Longer-term follow-up, incorporating objective anatomical imaging, is planned to validate these observations and assess the correlation between early HRM-based hernia persistence and future reflux recurrence.

## Data Availability

Deidentified datasets generated during the current study are available from the corresponding author on reasonable request. Not applicable; manuscript contains no identifiable personal data.
